# Production of full-length soluble *Plasmodium falciparum* RH5 protein vaccine using a *Drosophila melanogaster* Schneider 2 stable cell line system

**DOI:** 10.1038/srep30357

**Published:** 2016-07-26

**Authors:** Kathryn A. Hjerrild, Jing Jin, Katherine E. Wright, Rebecca E. Brown, Jennifer M. Marshall, Geneviève M. Labbé, Sarah E. Silk, Catherine J. Cherry, Stine B. Clemmensen, Thomas Jørgensen, Joseph J. Illingworth, Daniel G. W. Alanine, Kathryn H. Milne, Rebecca Ashfield, Willem A. de Jongh, Alexander D. Douglas, Matthew K. Higgins, Simon J. Draper

**Affiliations:** 1 The Jenner Institute, University of Oxford, Old Road Campus Research Building, Oxford, OX3 7DQ, UK; 2 Department of Biochemistry, University of Oxford, South Parks Road, Oxford, OX1 3QU, UK; 3 ExpreS^2^ion Biotechnologies, SCION-DTU Science Park, Agern Allé 1, Hørsholm DK-2970, Denmark

## Abstract

The *Plasmodium falciparum* reticulocyte-binding protein homolog 5 (PfRH5) has recently emerged as a leading candidate antigen against the blood-stage human malaria parasite. However it has proved challenging to identify a heterologous expression platform that can produce a soluble protein-based vaccine in a manner compliant with current Good Manufacturing Practice (cGMP). Here we report the production of full-length PfRH5 protein using a cGMP-compliant platform called ExpreS^2^, based on a *Drosophila melanogaster* Schneider 2 (S2) stable cell line system. Five sequence variants of PfRH5 were expressed that differed in terms of mutagenesis strategies to remove potential N-linked glycans. All variants bound the PfRH5 receptor basigin and were recognized by a panel of monoclonal antibodies. Analysis following immunization of rabbits identified quantitative and qualitative differences in terms of the functional IgG antibody response against the *P. falciparum* parasite. The antibodies induced by one protein variant were shown to be qualitatively similar to responses induced by other vaccine platforms. This work identifies *Drosophila* S2 cells as a clinically-relevant platform suited for the production of ‘difficult-to-make’ proteins from *Plasmodium* parasites, and identifies a PfRH5 sequence variant that can be used for clinical production of a non-glycosylated, soluble full-length protein vaccine immunogen.

*Plasmodium falciparum* parasites are the causative agent of the most severe form of human malaria, and the development of an effective vaccine remains a key strategic goal to aid the control, local elimination and eventual eradication of this disease. Next-generation vaccine strategies are now seeking to improve on the moderate levels of efficacy reported for the RTS,S/AS01 malaria vaccine which targets the pre-erythrocytic stages of the parasite’s lifecycle^1^. One leading strategy is to move towards a multi-antigen, multi-stage vaccine formulation, which will necessitate the development of effective vaccine components against the pathogenic asexual blood-stage of infection^2^ and the subsequent sexual-/mosquito-stages^3^. Blood-stage vaccines seek to induce antibodies against the merozoite form of the parasite that invades erythrocytes^2^, and could complement pre-erythrocytic immunity afforded by RTS,S/AS01, protect against disease severity and/or reduce transmission by accelerating the control and clearance of blood-stage parasitemia.

The development of an effective vaccine against the merozoite has proved exceptionally challenging, hindered by substantial levels of polymorphism in the most widely studied candidate antigens^4^ and redundant erythrocyte invasion pathways^5^. Moreover, the kinetic constraints imposed by such rapid erythrocyte invasion mean that extremely high concentrations of functional antibody are required to neutralize the parasite^6^. Progress in this arena, however, is being made with a new generation of merozoite antigen targets identified in recent years that exhibit relatively low levels of polymorphism and against which functional neutralizing antibodies can be raised by vaccination. Three of these targets form a complex, including the *P. falciparum* reticulocyte-binding protein homolog 5 (PfRH5)^7^, the PfRH5-interacting protein (PfRipr)^8^ and the cysteine-rich protective antigen (PfCyRPA)^9^; whilst another promising immunogen has included conserved regions III-V of the erythrocyte-binding antigen 175 kDa (PfEBA-175)^10^. Vaccine development efforts are currently the most advanced for PfRH5^11^.

Anti-merozoite vaccine studies have long relied on the standardized *in vitro* assay of growth inhibition activity (GIA)^12^, whereby purified IgG antibodies are tested against parasites cultured in human red blood cells (RBC) in the absence of other cell types. This assay has been shown to correlate with *in vivo* vaccine-induced protection in three non-human primate studies^13^,^14^,^15^. Preclinical studies using the assay of GIA have shown that antibodies raised by PfRH5 vaccination can cross-inhibit all *P. falciparum* lines and field isolates tested to-date^7^,^16^,^17^,^18^ and, secondly, that they can do so with higher efficiency than other historical target antigens (lower EC_50_ in terms of antigen-specific antibody concentration^16^). These studies have all used antibodies raised by full-length PfRH5 immunogens, given earlier reports using fragments of PfRH5 made in *Escherichia coli* that failed to induce functional antibodies^19^,^20^. In 2011 Crosnier *et al*. identified basigin (CD147) as the RBC receptor for PfRH5 with which it forms a critical non-redundant interaction during invasion^21^, in agreement with earlier reports that the PfRH5 gene is refractory to genetic deletion^19^,^22^. Basigin, like PfRH5, can also be targeted to block parasite invasion^23^,^24^,^25^, requiring even lower levels of antibody due to its constant exposure on the RBC surface, in contrast to the transient release on the apical merozoite surface of PfRH5. In the context of natural infection, PfRH5 appears to be a non-dominant target of naturally-acquired immune responses^7^,^26^, and thus the relatively high degree of PfRH5 sequence conservation is associated with low-level natural immune pressure, but also functional constraints linked to basigin binding and host RBC tropism^22^,^27^,^28^.

Generation of full-length PfRH5 protein proved particularly problematic, and thus the earliest promising results were achieved using viral vectored immunization^7^, whereby antigen is expressed *in situ* from virally-infected muscle cells^29^. Subsequently, production of full-length PfRH5 protein was reported using mammalian HEK293 cells^21^,^30^. Vaccination with both of these platforms led to signficant efficacy against a stringent heterologous strain blood-stage challenge in an *in vivo Aotus* monkey-*P. falciparum* challenge model^13^. Protection was strongly correlated with anti-PfRH5 serum IgG antibody concentration and *in vitro* functional GIA^13^, however, consistent with expectations^31^, the protein vaccine in strong adjuvant elicited higher antibody responses than the viral vectors. Consequently, although the first PfRH5 vaccine candidate to enter Phase Ia clinical trial is utilizing viral vectored delivery (Clinicaltrials.gov NCT02181088), there is strong momentum to develop a protein-based candidate to enable the highest possible antibody concentrations to be achieved following human immunization^32^.

Alongside the work in mammalian HEK293 cells, further progress has been made in recent years with reports of successful PfRH5 protein production from numerous other heterologous expression systems, including *E. coli*^18^,^33^, baculovirus-infected insect cells^34^,^35^ and a wheatgerm cell-free expression platform^36^, but notably not yeast-based systems. However, each of these reported approaches faces significant challenges for production of a clinically-compatible immunogen–including extremely low yield, the inclusion of C-terminal tags such as rat CD4 domains 3 and 4, production of insoluble protein within inclusion bodies, or lack of a scalable or compliant process for currrent Good Manufacturing Practice (cGMP) production of a clinical vaccine batch.

Here we report the production of soluble full-length PfRH5 protein using a cGMP-compliant platform called ExpreS^2^, based on a *Drosophila melanogaster* Schneider 2 (S2) stable cell line system^37^. Five sequence variants of full-length PfRH5 were expressed from stable cell lines and purified using a C-terminal hexa-histidine (His6) tag. The purification and biochemical analyses of these proteins are reported alongside a functional analysis of the antibodies induced by these vaccines following immunization of rabbits.

## Results

### Design of PfRH5 protein vaccine variant constructs

**Figure 1:**
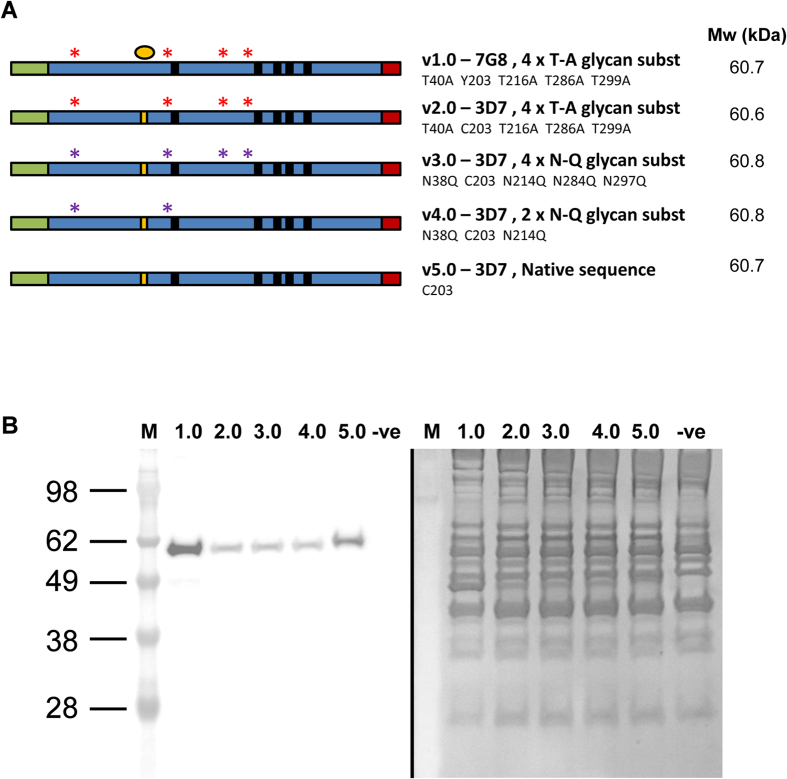
Polyclonal *Drosophila* S2 stable cell lines expressing PfRH5 protein variants. **(A)** Schematic of PfRH5 protein variants. All encoded from the N-terminus: a BiP insect signal peptide (green) followed by the ectodomain of PfRH5 (αα 26–526) (blue) followed by a C-terminal His6 tag (red). Protein v1.0 encoded the 7G8 strain sequence which differs from the 3D7 clone sequence by a single polymorphism Y203C (yellow). The other cysteine residues in PfRH5 are indicated by small black boxes (C224, C317, C329, C345 and C351). Amino acid substitutions to remove N-linked glycan sequons are indicated by red asterisks for threonine (T) to alanine (A), and purple asterisks for asparagine (N) to glutamine (Q). The predicted molecular weight (Mw) for each protein is also shown (based on the primary sequence only and not possible N-linked glycans). **(B)** Following transfection, stable polyclonal *Drosophila* S2 cell lines were generated encoding PfRH5 protein variants versions 1.0–5.0. 30 μL samples of culture supernatants from batch cultures were run on SDS-PAGE gels under reducing conditions and stained for total protein (right hand side) or Western blots performed with anti-Penta-His mAb (left hand side). Results are shown for each variant. M = molecular weight markers. −ve = S2 cell supernatant control.

We initially designed a series of vaccine constructs encoding the full-length ectodomain of the PfRH5 antigen (αα E26-Q526) (Fig. 1A). The rationale for these was based on previous experience with PfRH5 vaccines developed in other systems. Protein versions 1.0 and 2.0 were based on the 7G8 laboratory-adapted parasite line and the 3D7 clone of *P. falciparum* respectively, and all four putative N-linked glycosylation sequons (N-X-S/T) were mutated Thr to Ala–as performed for a previous PfRH5 protein vaccine produced in mammalian HEK293 cells and tested in rabbits^17^ and *Aotus* monkeys^13^. The only difference otherwise between these two proteins was the presence of the naturally occurring Y203C polymorphism^22^. The 7G8 sequence was initially included in case any complications arose with regard to protein expression and/or stability due to the presence of an unpaired buried cysteine^38^,^39^ in the 3D7 sequence. Excluding position 203, there are five cysteines in this construct. Crystal structures have since shown that four of these form two disulfide bonds (C224–C317 and C345–C351) while the fifth (C329) is unpaired but predominantly buried in a hydrophobic pocket between the three helices of the N-terminal half of PfRH5. Position 203 (either C or Y) is exposed on the surface of crystallized constructs of PfRH5, where it lies within the basigin binding site^35^,^40^.

We also made several constructs in which the glycosylation profile was modified. The version 3.0 sequence encoded the same 3D7 sequence, except that the four putative sites of N-linked glycosylation were instead mutated Asn to Gln. The version 4.0 sequence was identical to version 3.0, except that only two of the putative N-linked glycosylation sites were mutated (N38Q and N214Q). This construct was originally designed on the basis of NetNGlyc N-linked glycosylation prediction^41^, which suggested only these two sites were likely to be glycosylated by mammalian cells. This version 4.0 PfRH5 sequence was used for the development of viral vectored vaccines against full-length PfRH5, and elicited functional antibodies in rabbits and *Aotus* monkeys^7^,^13^. Chimpanzee adenovirus serotype 63 (ChAd63) and modified vaccinia virus Ankara (MVA) vectors encoding this sequence have since progressed to Phase Ia clinical trial in Oxford, UK (Clinicaltrials.gov NCT02181088). The final variant, version 5.0, encoded the native 3D7 PfRH5 sequence for comparison. All PfRH5 sequences were preceded at the N-terminus by the BiP insect leader sequence and finished with a His6 purification tag at the C-terminus. All constructs were codon-optimized for expression by *D. melanogaster*, and were cloned into the pExpreS^2^-1 expression plasmid that allows for Zeocin selection in transfected *Drosophila* S2 cells.

### Generation of polyclonal *Drosophila* S2 stable cell lines expressing PfRH5 protein variants

In order to assess PfRH5 protein expression by *Drosophila* S2 cells, transient transfections were initially performed. Plasmids encoding each of the variants were used to transfect cells as described in Methods, and 4 days later cell culture supernatants were harvested for analysis. Samples were analyzed by reducing SDS-PAGE followed by Western blot against the C-terminal His6 tag. All five variants were shown to express, with single bands visible by Western blot at the predicted size of approximately 60 kDa, however these were not identifiable by total protein staining within the protein-rich supernatant (Fig. S1A). Given the clear expression of soluble PfRH5 antigen by the *Drosophila* S2 cells, stable cell lines expressing each variant were subsequently generated over a 26 day period using Zeocin selection as described in Methods. Cell lines were frozen down and supernatants harvested for a similar analysis by Western blot as for the transient transfection. Each stable cell line was shown to produce the PfRH5 protein, with each antigen again visible only by Western blot at the expected size of approximately 60 kDa (Fig. 1B). Version 5.0 showed lower mobility than the other variants, likely due to the presence of N-linked glycan(s). An anti-PfRH5 quantification capture ELISA showed expression levels to be in the range of 5–30 μg/mL across the five cell lines (data not shown).

### Purification of PfRH5 protein variants from polyclonal *Drosophila* S2 stable cell line supernatants

**Figure 2:**
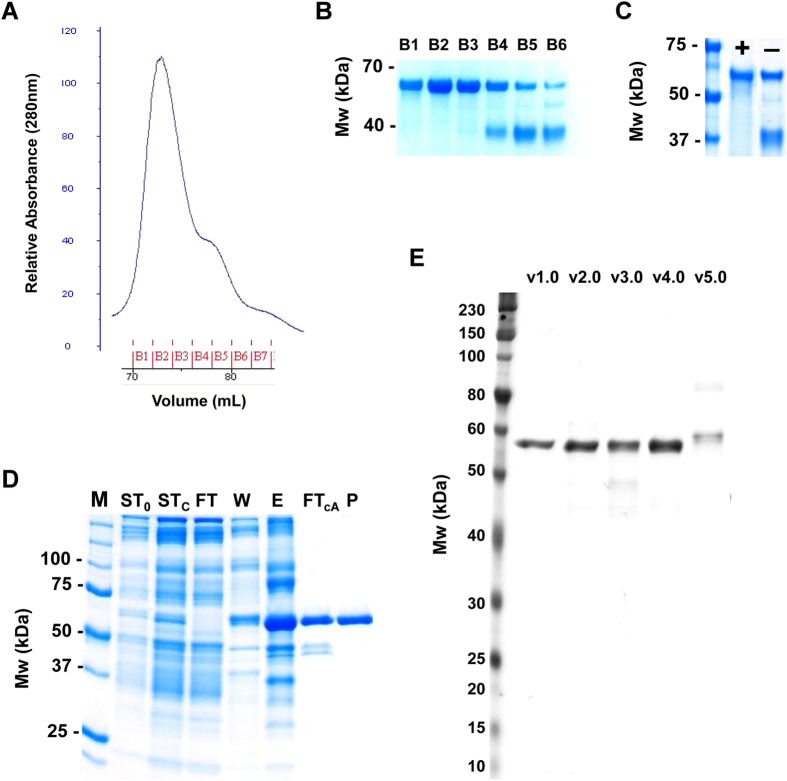
Purification of PfRH5 protein variants. **(A)** Size exclusion chromatography (SEC) curve of the Ni-IMAC peak fraction pool run on Superdex200 in 20 mM HEPES pH 7.4, 150 mM NaCl. The PfRH5 peak and the shoulder of contaminant 38 kDa *Drosophila* S2 protein are shown. **(B)** Coomassie gel analysis of SEC fractions. 10 μL of the SEC fractions B1–B6 were resolved on a 12% Tris-Glycine gel and proteins were stained with InstantBlue. **(C)** Coomassie gel analysis of pools of PfRH5 SEC peak fractions showing product purified in the presence (+) or absence (−) of the Con A 4B Sepharose purification step. Proteins were resolved on a 12% Tris-HEPES gel and stained with InstantBlue. **(D)** Coomassie gel analysis of a representative purification run of PfRH5 v1.0. Proteins were separated on a Bio-Rad Any kD TGX gel and stained with SimplyBlue SafeStain. M: molecular weight markers; ST_0_: starting material; ST_C_: starting material after TFF and buffer exchange; FT: flow-through from HisTrap column; W: HisTrap 40 mM Imidazole wash; E: Elution from HisTrap with 400 mM Imidazole; FT_cA_: flow-through from Con A column; P: final product after SEC. **(E**) Coomassie gel analysis of all purified PfRH5 protein variants run head-to-head (v1.0–v5.0). 1.25 μg each protein sample (DTT reduced and heat denatured) was separated on a Bio-Rad Any kD Criterion TGX gel and stained with InstantBlue.

Initial work focussed on the purification of PfRH5 v1.0 from four-day batch culture supernatants following expansion of the cell line in shaker flasks. Western blot analysis of supernatant under non-reducing conditions showed the bulk of the product to be monomeric, with a small mobility shift compared to reducing conditions–consistent with another report of PfRH5 protein made in *E. coli*^18^ (Fig. S1B). Clarified supernatant was subsequently concentrated and exchanged into IMAC equilibration buffer by TFF. The material was then purified by Ni-IMAC followed by a polishing step and final buffer exchange using SEC. The absorbance profile following SEC from these purification runs consistently revealed a major peak followed by a small shoulder (Fig. 2A). Analysis of the corresponding elution fractions confirmed a protein of approximately 60 kDa (later shown to be PfRH5 v1.0) present in the major peak, with the shoulder containing the same protein plus a major contaminant of approximately 38 kDa (Fig. 2B). Analysis of the contaminant 38 kDa protein by mass spectrometry revealed this to be the *Drosophila* windpipe (wdp) glycoprotein^42^. The predicted extracellular domain of wdp contains four putative leucine-rich repeat (LRR) motifs that are found in numerous proteins and function in mediating protein–protein interactions.

Given a significant proportion of the PfRH5 protein was being lost due to co-elution with this contaminant, numerous column purification strategies were explored to prevent co-purification of PfRH5 with wdp. The strategy that yielded the highest degree of success was to include a Con A purification step following the Ni-IMAC and prior to SEC. This step removed the wdp contaminant, presumably due to interaction of the Con A lectin with the wdp glycoprotein but not the PfRH5 protein (Fig. 2C). The final purification protocol using TFF–Ni-IMAC–Con A–SEC yielded pure PfRH5 v1.0 protein as shown by Coomassie staining (Fig. 2D).

Subsequently, the PfRH5 protein variants v1.0–v3.0 were all purified using this protocol. Variants v4.0 and v5.0 which potentially contained glycosylated PfRH5 could not be purified using the Con A lectin step, meaning lower overall yields were achieved due to the greater loss of PfRH5 protein during the SEC polishing step; although it should be noted that pure PfRH5 could still be produced from the elution fractions lacking the wdp contaminant (Fig. 2B). Final analysis of each variant by SDS-PAGE and Coomassie staining revealed protein with >95% purity, and v5.0 protein again ran at a slightly higher molecular weight than the others (Fig. 2E). Overall, however, the process yields were low, averaging less than 5% on most runs with the heaviest losses taken during the Ni-IMAC. Example data for v1.0 protein are shown in Table 1.

### Biochemical characterization of PfRH5 protein variants

**Figure 3:**
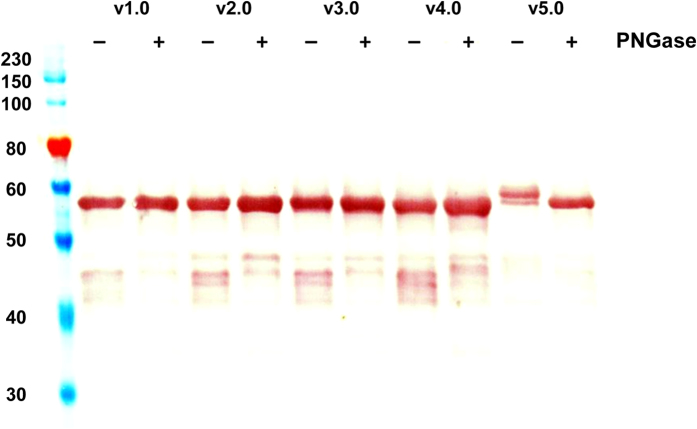
Assessment of PfRH5 protein variant glycosylation. The glycosylation state of the recombinant PfRH5 variant proteins was analyzed by SDS-PAGE following PNGaseF treatment. Purified PfRH5 variant proteins were either untreated (−) or digested (+) with N-glycosidase PNGaseF. 0.5 μg of each sample was run on SDS-PAGE prior to Western blot.

The glycosylation state of the recombinant PfRH5 variant proteins was analyzed by SDS-PAGE following PNGaseF treatment. The shift in mobility of the v5.0 protein with all four potential sites of N-linked glycosylation intact confirmed that it was glycosylated (Fig. 3). There was no evidence that the v4.0 protein was glycosylated, indicating that the NetNGlyc prediction was correct and that one or both of the N-X-T sequons starting at αα positions N38 and N214 are the ones glycosylated in the v5.0 protein.

**Figure 4:**
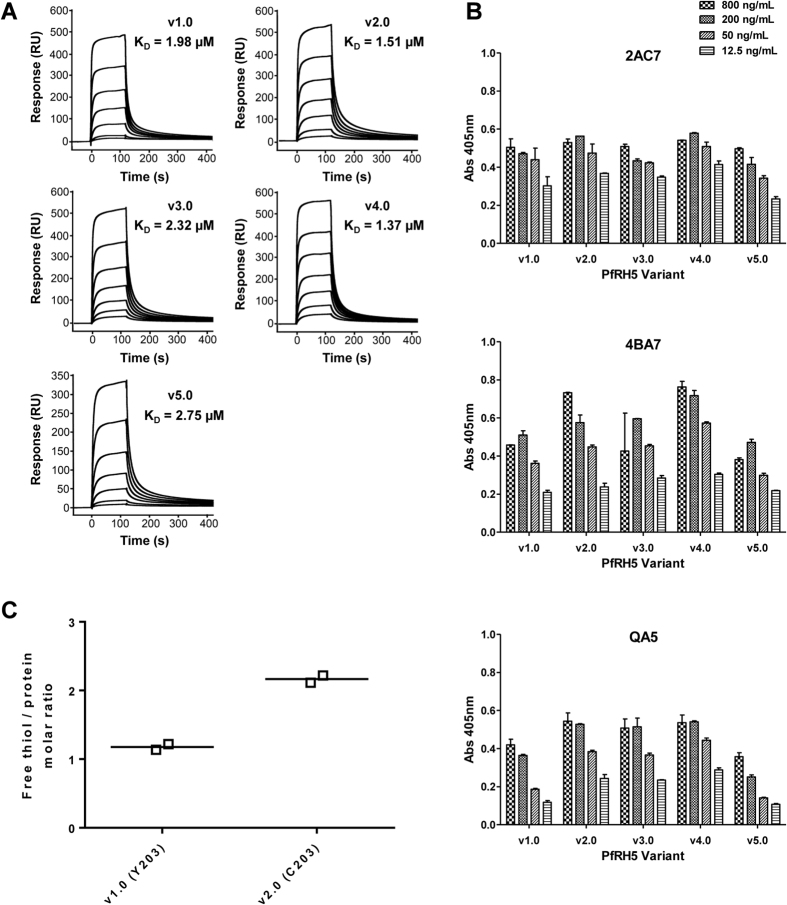
Characterization of PfRH5 protein variants. **(A)** Surface plasmon resonance analysis of the interaction of each PfRH5 protein variant (v1.0–v5.0) with basigin. **(B)** Capture ELISA using a panel of PfRH5-specific mAbs. Each mAb (2AC7, 4BA7 and QA5) was coated to the plate at a concentration of 5 μg/mL, and each PfRH5 protein variant was tested for binding using a dilution series of protein ranging from 800 ng/mL to 12.5 ng/mL. Each sample was tested in triplicate for each concentration. Bars show the median plus range. Results using five other mAbs are shown in Fig. S2. **(C)** Free sulfhydryl groups were estimated for v1.0 and v2.0 protein variants using Ellman’s reagent in the presence of 6 M guanidine-HCl. Median and replicate results are shown.

The ability of each full-length protein variant to bind to recombinant basigin was subsequently analyzed by SPR. Each variant bound with an affinity close to the expected K_D_ of 1–2 μM (Fig. 4A)^13^,^21^. The ability of each variant to be recognized by a panel of eight previously characterized mouse mAbs^23^ was also assessed by ELISA (Figs 4B and S2). All five variants bound to the mAbs comparably, confirming the presence of each epitope in each protein. Finally we compared the v1.0 and v2.0 proteins in terms of free thiol content for denatured protein (Fig. 4C), given the single C203Y polymorphism that differs between these two constructs (Fig. 1A). These data confirmed the presence of one free thiol (C329) in the v1.0 protein, and two free thiols in the v2.0 protein (C203 and C329).

### Immunological analysis of PfRH5 protein variants

**Figure 5:**
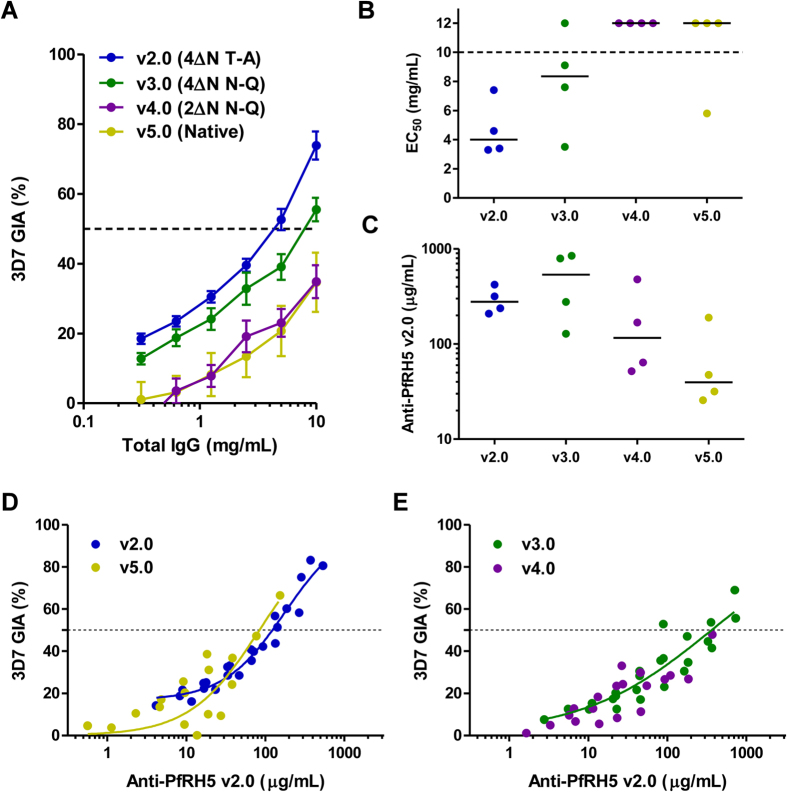
Immunological analysis of PfRH5 protein variants. **(A)** GIA against 3D7 clone parasites versus total IgG concentration, with lines connecting data for each group of vaccinated rabbits (*n* = 4/group). Each point is the mean ± SEM for each group with each rabbit tested in duplicate in two independent experiments, i.e. *n* = 8. **(B)** GIA EC_50_ in terms of total IgG concentration (mg/mL). Points show the mean result for each rabbit tested in duplicate in two independent experiments. Eight rabbits did not achieve 50% GIA at the highest tested IgG concentration, i.e. EC_50_ > 10 mg/mL and these are all plotted arbitrarily as 12 mg/mL. Median lines are shown. **(C)** Anti-PfRH5 v2.0 ELISA results are shown quantified in terms of μg/mL. Individual and median results are shown for each group. **(D)** Dose-response curve fitted to all GIA versus anti-PfRH5 v2.0 antigen-specific antibody concentration data (all IgG dilutions for each rabbit immunized with v2.0 or v5.0 are shown). Dashed horizontal line indicates 50% GIA. Each GIA value is the mean of two independent experiments with triplicate wells in each. Non-linear least squares regression line is shown; r^2^ = 0.95 for v2.0 and 0.72 for v5.0, *n* = 24 for both. **(E)** Same as panel D, showing data for rabbits immunized with v3.0 and v4.0 proteins. Regression line is only shown for v3.0; r_2_ = 0.82, *n* = 24; and not v4.0 given no animal achieved >50% GIA at any tested dilution.

In order to test the immunogenicity and functional activity of vaccine-induced antibodies, we immunized rabbits with protein versions 2.0–5.0. We chose to focus on these four variants given they are based on the 3D7 clone of malaria commonly used for CHMI proof-of-concept efficacy testing in humans, including for use in the blood-stage challenge model^43^. Rabbits were immunized three times with 20 μg antigen formulated in Freund’s adjuvant prior to serum harvest. IgG was subsequently purified and assessed for *in vitro* GIA using a single-cycle assay. Notably, protein v2.0 exhibited the highest levels of GIA with a median EC_50_ of 4 mg/mL total IgG (Fig. 5A,B), similar to full-length PfRH5 protein vaccines produced in mammalian cells and *E. coli*^17^,^18^. Protein v3.0 was approximately 2-fold less effective with a median EC_50_ of 8.4 mg/mL total IgG, whilst v4.0 and 5.0 failed to achieve 50% GIA even at the highest concentration tested (10 mg/mL) although modest GIA of around 30–40% was still observed.

The differences in GIA seen here could be due to differences in anti-PfRH5 antibody quantity and/or quality. To address this, each rabbit serum sample was initially tested for anti-PfRH5 IgG responses by ELISA against v2.0 protein–this variant was chosen because it lacked N-linked glycans. Following ELISA, responses were roughly comparable between v2.0 and v3.0 immunized animals, although a larger spread was seen in the v3.0 group. Median responses of 278 and 535 μg/mL PfRH5-specific IgG were observed respectively (Fig. 5C). Notably, responses were lower in v4.0 and v5.0 immunized rabbits with medians of 116 and 40 μg/mL PfRH5-specific IgG, respectively. These immunogenicity data suggested v5.0 protein, and to a lesser extent v4.0, tended to be less immunogenic in agreement with the GIA results.

However, these ELISA data using sera did not account for possible changes due to IgG purification or IgG concentration normalization prior to performing the GIA assay. In order to address this in further detail, the concentration of rabbit total IgG in each serum sample was first assayed by ELISA (median = 12.6 mg/mL; range 7.9–19.6 mg/mL; *n* = 16). Subsequently, the ELISA result for each serum sample (Fig. 5C) was normalized according to the serum IgG concentration to give the μg/mL anti-PfRH5 IgG concentration per mg total IgG. The GIA data for each individual rabbit (Fig. 5A) were then replotted against the concentration of anti-PfRH5 IgG present in each purified IgG sample tested in the assay (Fig. 5D,E). These data showed that GIA was associated with anti-PfRH5 IgG concentration, with a typical sigmoidal relationship, as observed in numerous other studies with other antigens^44^,^45^. Notably, the fitted curves generated with v2.0 and v5.0 were similar (Fig. 5D) and in contrast to the data for v3.0 and v4.0 where the slope of the curve was less steep (Fig. 5E). The antigen-specific EC_50_ was interpolated from the data for each individual rabbit–this was calculated for nine of the rabbits that reproducibly achieved >40% GIA in the original assay (Fig. 5A). These data for v2.0 showed a median EC_50_ of 129 μg/mL (95% CI 80.7–186.7 μg/mL) anti-PfRH5 IgG (Fig. S3A). For the interpretation of these data, it should also be noted that although v2.0 protein was used for these serological analyses, small numbers of polymorphic residues that differ between PfRH5 allelic variants would be unlikely to have a gross effect on ELISA cross-reactivity, as reported in a previous study^17^. Moreover, even if some responses were not measured by ELISA for the v3.0–v5.0 sera, this would have led to under-reporting of these antibody levels and thus an over-estimation of antibody-specific IgG functional quality, suggesting that the differences seen here between these and the v2.0 sera could be even larger.

Overall these results suggested that the antibody responses induced by v2.0 and v5.0 proteins were qualitatively similar, and thus v5.0 performed less well in the GIA assay due to substantially reduced immunogenicity as seen in the serum ELISA (Fig. 5C). In contrast, v3.0 and v4.0 proteins tended to induce an antibody response of inferior quality (Fig. S3A), likely explaining why v3.0 performed less well in the GIA assay in comparison to v2.0, despite comparable, or even better, quantitative immunogenicity as seen in the ELISA.

### Comparison with PfRH5 vaccines produced in other platforms

The antigen-specific EC_50_ reported above for the v2.0 protein produced in S2 cells was higher than that which we previously reported following immunization of rabbits with adeno- and pox-viral vectored vaccines that express full-length PfRH5^16^–in this case, median EC_50_ = 64 μg/mL (95% CI of mean 46–101 μg/mL, *n* = 5). Although there was overlap in the 95% CI between these two studies, we sought to confirm whether or not the S2 cell-produced v2.0 protein elicited a similar quality of antibody response to other vaccine platforms. We repeated the rabbit immunization with viral vectors expressing full-length PfRH5 (a construct similar to v4.0 reported here) and also immunized rabbits with a full-length PfRH5 protein produced in mammalian HEK293E cells (a construct similar to v2.0 reported here but with C-terminal tags including rat CD4 domains 3 and 4 and His6, called PfRH5-Cd4-His6) (Fig. S3B)–both of these vaccine platforms have been previously reported and tested in *Aotus* monkeys^13^. We also re-analyzed the historical sera from the five rabbits immunized in the same manner with the viral vectors^16^. These data showed that the rabbits in the current study had a comparable quality of response, irrespective of how they were immunized across the three platforms–S2 cell protein, HEK293 protein or viral vectors (*P* = 0.09, Kruskal-Wallis test with Dunn’s correction for multiple comparisons) (Fig. S3C). In our previously reported study, antigen-specific IgG concentrations were measured by a SPR technique called calibration-free concentration analysis^16^. Here, using the affinity-purified IgG ELISA standard method^44^, we obtained a very similar result: median EC_50_ = 48 μg/mL (95% CI of mean 29–96 μg/mL, *n* = 5). These responses were significantly lower than those seen in the rabbits immunized in the current study with the same viral vectors (*P* = 0.02, Mann Whitney U test), suggesting the quality of vaccine-induced antibody response can vary and future comparisons need to be conducted within the same rabbit study.

## Discussion

The production of a clinically-suitable soluble full-length PfRH5 protein immunogen using a cGMP-compliant platform has proved challenging. Here we report substantial progress in this regard, through the use of the ExpreS^2^ platform based on a *Drosophila* S2 stable cell line system^37^. Five sequence variants of PfRH5 were initially designed, cloned and transfected into the *Drosophila* S2 cells and polyclonal stable cell lines generated that secreted full-length protein into the supernatant at the expected size of approximately 60 kDa. Our constructs replaced the native parasite signal peptide with the insect BiP leader sequence, and initiated at position E26 comparable to other reported proteins that have started at positions S24, F25 or N27^18^,^30^,^35^, running through to the final amino acid Q526.

Although soluble, monomeric PfRH5 was easily detectable in the supernatants by Western blotting, purification of the protein from the protein-rich supernatant proved challenging, despite the presence of the C-terminal His6 tag to enable Ni-IMAC. Depending on the construct, up to four purification steps were required, resulting in high >95% purity but a low overall process yield, typically <5% recovery. Consequently this purification strategy will not be suitable for scale-up and clinical biomanufacture of the PfRH5 protein vaccine. Further measures to improve overall process yields have since been explored and will be reported elsewhere, including the use of alternative C-terminal tags for improved affinity chromatography and the isolation of high-expressing stable monoclonal cell lines in contrast to the polyclonal pools reported here [Jin *et al*., in preparation]. As the *Drosophila* S2 cell line platform becomes more widely studied and used for clinical development it may also be possible to engineer out any genes that encode problematic contaminants, such as wdp which was identified here.

Each PfRH5 protein variant, including v1.0 and v2.0 differing by the C203Y polymorphism, bound recombinant basigin with an affinity close to the expected K_D_ of 1–2 μM, in agreement with the original reports for protein produced in mammalian HEK293 cells^13^,^17^,^21^, but dissimilar to the report of another protein produced in baculovirus-infected Hi-5 insect cells, in which the presence of oligomeric forms of PfRH5 may have contributed to a higher apparent K_D_^35^. The *Drosophila* S2 cell produced proteins were also all recognized by a panel of eight previously characterized mouse mAbs known to bind linear and conformational epitopes, including those within the basigin binding site^23^,^40^. Biochemical analysis of free thiol groups using Ellman’s reagent confirmed the single polymorphism C203Y that differed between the v1.0 and v2.0 proteins.

**Figure 6:**
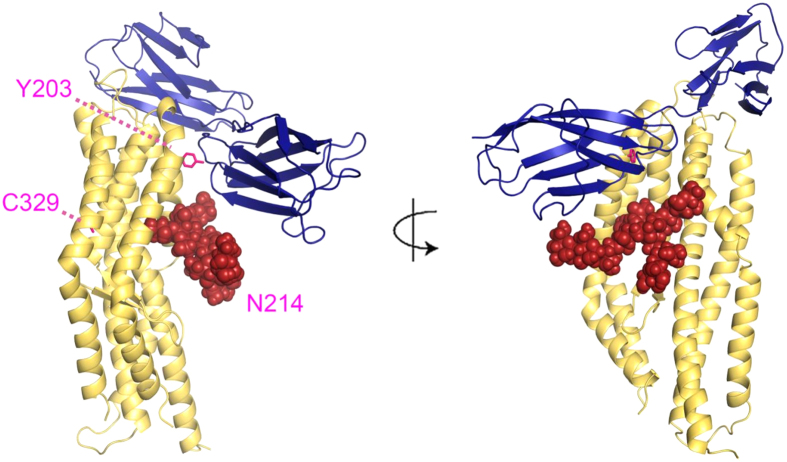
Structural features on PfRH5. Structural features are outlined on the crystal structure of PfRH5 (αα K140–K247 + N297–Q526, yellow) bound to the two Ig domains of the basigin receptor (blue)^40^. The putative N-linked glycan is shown in red at position N214 with two N-acetyl glucosamines and nine mannose residues–the largest type of glycan found on the insect cell expressed proteins^60^. The other site which may be glycosylated, N38, is within the disordered N-terminal region of PfRH5 which is not shown in the structure. The buried cysteine at position C329 and the C203Y polymorphism are highlighted in pink.

We also assessed the glycosylation profile of the protein variants. While *Plasmodium* parasites rarely attach N-linked glycans to proteins^46^, insect and mammalian cells tend to attach large and often complex glycans. This may be problematic because N-linked glycans could potentially mask neutralizing epitopes on the antigen^47^. Thus in order to remove the four sites of potential N-linked glycosylation (N-X-S/T) we assessed mutation of Thr to Ala, versus Asn to Gln. None of these eight mutated residues are known positions of natural polymorphism in PfRH5, but the introduction of four mutations mirrors the greatest level of divergence observed between the 3D7 clone PfRH5 sequence and that reported for 227 field isolates^16^,^17^,^22^. Importantly, none of these sites are in the known basigin-binding region^40^ (Fig. 6) thus potentially minimizing impact on neutralizing epitopes, and notably all eight of our mouse mAbs bound each PfRH5 protein variant in a comparable manner suggesting these specific epitopes were not disrupted. However, N-linked glycans are often essential to protein folding in the endoplasmic reticulum of mammalian cells, and mutation in critical residues of PfRH5 could also have a detrimental impact on the immunogen conformation. Experience with PfRH5 in the mammalian HEK293 cell system suggests this is unlikely, whereby a protein with these sites mutated Thr to Ala was functional and showed improved expression levels over native sequence^21^,^30^. Occasionally, mutation of N-linked glycosylation sites can impinge on the ability of an antigen to elicit functional antibodies, as seen with Pfs48/45 when delivered by genetic immunization^48^. In contrast, various other *Plasmodium* antigens have been demonstrated to elicit functional immune responses following genetic and protein immunization without modification to remove N-glycan sequons^29^,^49^,^50^,^51^,^52^ or prevent other forms of post-translational modification^53^, whereas in one reported case removal of N-linked glycans had a positive impact on *in vivo* vaccine efficacy^42^.

In the case of PfRH5, v5.0 protein with all four potential N-linked glycosylation sequons intact was glycosylated with no evidence that the v4.0 protein was glycosylated. These data supported the NetNGlyc prediction that one or both of the sequons starting at N38 and N214 are glycosylated (and not N284 or N297). N284 is present in the disordered loop between helices 2 and 3 and was not reported in either structure; whilst N297 is buried^35^–consistent with the absence of glycosylation in the v4.0 protein. N38 is present in the disordered N-terminal region of PfRH5 that is processed away from the molecule in cultured parasite lines to leave a product of approximately 45 kDa^8^,^19^,^20^,^22^,^35^,^40^. The RB3 mouse mAb binds this N-terminal region, but peptide analysis indicated N38 is not present in the epitope^23^, and so further mutagenesis studies would be required to definitively conclude whether this asparagine is glycosylated in the *Drosophila* S2 cell expression system. In the reported crystal structures^35^,^40^ residue N214 is surface exposed and could be glycosylated (Fig. 6). However, consistent with our mAb- and basigin-binding data for the v5.0 protein, glycosylation of N214 would likely have minimal impact in terms of glycan masking, given this residue is not present in the known epitopes of any neutralizing mAb^23^,^40^ nor is it present in the basigin binding site. Nevertheless, following immunization of rabbits, the glycosylated v5.0 protein elicited the lowest antibody concentrations. The presence of the N-linked glycan(s) thus appeared to have a negative impact on PfRH5 immunogenicity, although the induced antibodies appeared qualitatively similar to v2.0, suggesting that if the N-linked glycan(s) did mask epitopes then this was not detrimental. Interestingly, both the v3.0 and v4.0 proteins appeared to elicit a qualitatively inferior response in comparison to v2.0, suggesting a negative impact of Asn to Gln mutation, at least for residues N38Q and N214Q (the mutations common to both proteins). Version 2.0 protein, with four Thr to Ala mutations, performed well in terms of both quantitative and qualitative immunogenicity, eliciting the highest levels of GIA. However it should also be noted that any possible negative impact of these four Thr to Ala mutations remains unknown, given a non-glycosylated native sequence protein comparator could not be produced in this system. Overall, even with the guidance of protein structure, studying the possible outcomes of N-linked glycan site mutation and glycan masking on vaccine efficacy will likely remain an empirical endeavour.

In the final analysis, we compared the quality of the antibodies elicited by the v2.0 protein produced in *Drosophila* S2 cells, to those induced by immunization with viral vectors, or a protein produced in mammalian HEK293 cells–both of which have been previously assessed for *in vivo* efficacy in the *Aotus* monkey model^13^. Qualitatively, each platform performed in a similar manner with median EC_50_ antigen-specific antibody concentrations in the region of 100–170 μg/mL. Notably this was 2–3 folder higher than our previously reported study using exactly the same viral vectored vaccines in rabbits^16^. We re-analyzed these historical sera to rule out any changes in methodology over time, and confirmed our previous results. These data would suggest that different colonies of rabbits may perform differently over time, and thus future comparative analyses of antibody quality need to be undertaken in the same rabbit study. These data also highlighted qualitative differences between immunization with viral vectors and *Drosophila* S2 cell protein–in this case the vectors encoded a PfRH5 protein with the v4.0 sequence but this did not underperform in comparison to the HEK293 protein and *Drosophila* S2 cell protein both of which possessed the v2.0 sequence. These data suggest *in situ* antigen production from the immunized muscle cells led to some improvement in immunogen quality, and are also in agreement with our previous *Aotus* study whereby the viral vectors and HEK293 cell-produced protein produced responses with no obvious qualitative differences and strong associations with protection irrespective of immunization platform^13^.

In summary, the *Drosophila* S2 cell platform is receiving increasing recognition as a cGMP-compliant platform suited for the production of ‘difficult-to-make’ proteins–including the pregnancy malaria-associated antigen VAR2CSA^54^ and the envelope proteins for dengue virus and West Nile virus vaccines^55^. The PfRH5 v2.0 protein variant reported here provides the sequence for a clinically-suitable, non-glycosylated, soluble full-length PfRH5 protein immunogen. Once an improved and scalable purification strategy has been defined, this will enable the clinical production of a first-generation PfRH5 protein vaccine for assessment in Phase I/II clinical trials.

## Materials and Methods

### Design and cloning of PfRH5 protein variant constructs

Five synthetic genes were designed based on the full-length PfRH5 amino acid (αα) residues E26-Q526 from the 7G8 laboratory-adapted parasite line or the 3D7 clone of *P. falciparum*. The rationale and details of each construct are explained in Fig. 1A and the Results. Each gene was codon-optimized for expression in *D. melanogaster* (GeneArt, Life Technologies); and was flanked by an EcoRI site and Kozak sequence (GCC ACC) at the 5′ end, and a NotI site at the 3′ end. All constructs contained an N-terminal 18 αα BiP insect signal peptide (MKLCILLAVVAFVGLSLG) and a C-terminal His6 tag, and were subcloned into the pExpreS^2^-1 plasmid allowing for Zeocin selection (ExpreS^2^ion Biotechnologies, Denmark).

### Generation of polyclonal *Drosophila* S2 transient and stable cell lines

The ExpreS^2^ *Drosophila* S2 cell line was used for all work (ExpreS^2^ion Biotechnologies, Denmark). The day before transient transfection, cells were split by centrifugation (450 × *g*, 3 min) and resuspended to 8 × 10^6 ^cells/mL in EX-CELL 420 serum-free medium for insect cells (Sigma) in 250 mL shake flasks and incubated at 115 rpm at 25 °C. On the day of transfection, cells were split as before to 5 × 10^6 ^cells/mL. For each transfection, 200 μL ExpreS^2^ Insect-TRx5 transfection reagent (ExpreS^2^ion Biotechnologies, Denmark) was added to 8 mL cell suspension and gently mixed, before addition of 50 μg plasmid DNA, gentle mixing and then a 5 min rest. Cells were then incubated at 25 °C and 200 rpm in a 50 mL centrifuge tube with vent cap. On day 4 the cells were harvested and the supernatant saved for analysis.

To generate polyclonal stable cell lines, cells were split as for transient transfection on day 0 except they were resuspended to 5 × 10^6 ^cells/mL in EX-CELL 420 media + 10% fetal bovine serum (FBS). The following day, cells were resuspended to 2 × 10^6 ^cells/mL in EX-CELL 420 media, and 5 mL cell suspension was transferred to a T25 flask. 250 μL ExpreS^2^ Insect-TRx1 transfection reagent (ExpreS^2^ion Biotechnologies, Denmark) was added before gentle mixing and then addition of 12.5 μg plasmid DNA followed by gentle mixing. Flasks were then incubated at 25 °C for 2–4 h before addition of 1 mL FBS. On day 2, 1500 μg/mL Zeocin was added for selection. From day 4 through 24 post-transfection, each flask was inspected every 3–4 days and diluted down to approximately 1 × 10^6 ^cells/mL by replacing of old media with fresh EX-CELL 420 media + 10% FBS + Zeocin. On day 24, 6 mL cell culture from each flask was transferred to a T75 flask and 4 mL of EX-CELL 420 media + 10% FBS + Zeocin was added. After the cells had recovered, 5 mL more of EX-CELL 420 media + 10% FBS + Zeocin were added. On day 26, the cell suspension (15 mL in total) was transferred directly to a 125 mL shake flask with addition of 10 mL EX-CELL 420 media + 10% FBS. The cells were then passaged once by centrifugation to remove any residual Zeocin, before freezing in 1 mL of 40% EX-CELL 420 + 50% FBS + 10% DMSO. In a separate 125 mL shake flask the cells were passaged twice in EX-CELL 420 without FBS before a sample of the supernatant was taken for analysis.

### SDS-PAGE and Western blots

For analysis of stable and transient transfections (Figs 1B and S1A), samples of supernatant were prepared in 10X Bolt Sample Reducing Agent and 4X Bolt LDS Sample Buffer, and heat treated for 5 min at 85 °C. Samples were run by SDS-PAGE on a Bolt 10% Bis-Tris Plus gel in Bolt MES SDS running buffer at 165 V for 35 min. The gels were stained with SimplyBlue SafeStain for total protein, or transferred to a nitrocellulose membrane with an iBlot Transfer Stack. Blots were stained using the Penta·His HRP Conjugate Kit (Qiagen) according to the manufacturer’s protocol and detected with Novex ECL Chemiluminescent Substrate Reagent Kit. SeeBlue Plus2 Pre-Stained Marker (Life Technologies) was used. For analysis of sample purity by SDS-PAGE (Fig. 2B–E), 2 μg protein was heated to 95 °C for 5 min in Laemmli sample buffer containing 2.5% β-mercaptoethanol. Electrophoresis was performed on a Criterion Any kD TGX gel (Bio-Rad Laboratories, Herts, UK) at 200 V for 42 min using Tris-Glycine-SDS running buffer. The gels were then stained with InstantBlue (Expedeon, Cambridge, UK). Purity was determined from gel band density using ImageJ software.

### Recombinant PfRH5 protein purification

Stably transfected *Drosophila* S2 cells expressing His6-tagged PfRH5 variants were maintained in suspension in Erlenmeyer shake flasks (Corning) at 25 °C in an Innova 44 shaking incubator (New Brunswick Scientific) set at 115 rpm. Cells were cultured in EX-CELL 420 serum-free medium supplemented with 100 IU/mL penicillin and 100 μg/mL streptomycin (Sigma). Every three to four days the cell density was determined manually by counting in the presence of trypan blue (Sigma) and the cells were subcultured to 8 × 10^6^/mL alternately by dilution, or centrifugation and resuspension in fresh media.

Recombinant PfRH5 protein was produced from clarified four-day batch culture supernatant following expansion to the desired culture volume, typically 2 L. Prior to Nickel Immobilized Metal Affinity Chromatography (Ni-IMAC), the clarified supernatant was concentrated and buffer-exchanged using a LV Centramate lab Tangential Flow Filtration (TFF) system (PALL) fitted with two Omega T-series cassettes with 10 kDa cutoff membrane. Supernatant proteins were concentrated 15-fold prior to a 10-fold buffer exchange (v/v) with IMAC equilibration buffer: 20 mM sodium phosphate, pH 7.4; 250 mM NaCl; 20 mM Imidazole; 0.005% Tween-20. Following TFF and buffer-exchange, concentrated supernatant was snap-frozen in liquid nitrogen and stored at −80 °C until processed.

Recombinant PfRH5 was subsequently purified from buffer-exchanged and concentrated S2 cell supernatant using a three-step method on an AKTApurifier 100 system (GE Life Sciences). First, Ni-IMAC was performed using a 5 mL HisTrap FF column (GE Life Sciences) equilibrated with IMAC equilibration buffer. A 10 column volume wash with 40 mM Imidazole buffer removed proteins bound non-specifically, and a step to 400 mM Imidazole was employed for elution. Peak fractions from the Ni-IMAC elution were pooled and further purified on a 5 mL HiTrap Concanavalin A (Con A) 4B column (GE Life Sciences) equilibrated with Ni-IMAC elution buffer. Contaminant S2 proteins in the IMAC elution peak bound to the Con A column while recombinant PfRH5 was harvested from the flow-through and concentrated on an Amicon Ultra-15 centrifugal filter unit with 10,000 NMWL (Merck Millipore). Final polishing and buffer exchange was achieved by Size Exclusion Chromatography (SEC) on Superdex200 16/60 (GE Life Sciences) in 20 mM HEPES pH 7.4, 150 mM NaCl. SEC fractions containing pure PfRH5 were pooled and concentrated to 0.5–1 mg/mL as described above, snap-frozen in liquid nitrogen and stored at −80 °C. Protein concentration was determined by spectrophotometry (Nanodrop, Thermo Fisher Scientific, UK).

For the rabbit immunization study, a batch of PfRH5 protein was also produced in mammalian cells. This construct, called PfRH5-Cd4-His6, has been previously reported^13^ and is shown in Fig. S3B. Two litres of suspension HEK293E cells were transiently transfected with plasmid (kindly provided by Gavin Wright, Wellcome Trust Sanger Institute, UK) as previously described^13^ and culture supernatants were harvested after four days, before concentration 20-fold and subsequent exchange into PBS using a Pellicon 3 TFF system (Millipore, Herts, UK). Purification consisted of a cobalt-based IMAC–Hitrap TALON crude (GE Healthcare, Bucks, UK) and a SEC–HiLoad 16/600 Superdex 200 PG (GE Healthcare, Bucks, UK). The purified protein was quantified by Nanodrop and stored at −80 °C until further use.

### Anti-PfRH5 quantification ELISA

Maxisorp plates (Nunc-Immuno) were coated at 4 °C overnight in bicarbonate buffer with the anti-PfRH5 mouse mAb 2AC7^23^ at 5 μg/mL. Plates were washed with PBS/0.05% Tween-20 (PBS/T), blocked in Casein Blocker in PBS (Thermo Scientific) for 1 h at room temperature (RT), and washed again prior to addition of diluted PfRh5 protein samples in duplicate. Serially diluted pure PfRH5 v1.0 in the range 200 ng/mL to 1.56 ng/mL was added to each plate to generate a standard curve. Plates were incubated at RT for 2 h and then washed prior to addition of rabbit serum from animals immunized with PfRH5 (3D7) viral vectored vaccine^7^ diluted at 1:1000 in Casein Blocker. After a further 1 h incubation and subsequent wash, alkaline phosphatase-labelled goat anti-rabbit IgG (whole molecule) (Sigma) was added to each well at a 1:5000 dilution in Casein Blocker and plates were left to incubate for a final hour. Bound antibodies were detected by addition of *p*-nitrophenyl phosphate substrate (Sigma) diluted in diethanolamine buffer (Thermo Scientific). The optical density at 405 nm (OD_405_) was read using an ELx800 microplate reader (BioTek, UK) and a four parameter fit was generated from standard curve values with Gen5 ELISA software v1.10 (BioTek, UK). This curve was used to convert the absorbance of diluted PfRH5 samples into concentration of PfRH5 protein.

### Mass spectrometry

1.8 μg of concentrated protein from an SEC fraction containing PfRH5 and the 38 kDa S2 cell contaminant was resolved on a 12% precast polyacrylamide gel (Thermo Scientific) with Tris-HEPES-SDS running buffer (Thermo Scientific) according to the manufacturer’s directions. Proteins were visualized with SimplyBlue SafeStain (Invitrogen) and the 38 kDa protein band was excised for trypsin digestion and mass spectrometry as described in^56^. Mass spectrometry data were analyzed using Mascot (Matrixscience, London, UK).

### Characterization of protein glycosylation

The presence of glycosylation on the PfRH5 variant proteins was determined using the PNGase F kit (New England Biolabs, Herts, UK) under denaturing reaction conditions following the supplied protocol. Briefly, 2 μg protein was first heated to 95 °C for 5 min in the Glycoprotein Denaturing Buffer containing 0.5% SDS and 40 mM DTT, then chilled on ice before the addition of 1% NP-40, GlycoBuffer 2, and PNGase F, and then incubated at 37 °C for 1 h. Reaction products were separated on a Criterion Any kD TGX gel (Bio-Rad Laboratories, Herts, UK) using Tris-Glycine-SDS running buffer as previously described, and subsequently transferred to nitrocellulose membrane using a Trans-Blot Turbo Transfer System (Bio-Rad Laboratories, Herts, UK). Detection of PfRH5 proteins was performed using full-length PfRH5-reactive rabbit serum^7^ as the primary antibody, and alkaline-phosphatase labelled donkey anti-rabbit (Jackson ImmunoResearch, Suffolk, UK) as the secondary antibody. Blots were developed using BCIP/NBT (Sigma-Aldrich, Dorset, UK). The Western blot of the v1.0 PfRH5 stable cell line supernatant in Fig. S1B used the same methodology but with reducing and non-reducing conditions.

### mAb ELISA

The generation of eight PfRH5-specific mouse mAbs has been previously described^23^. Purified mAb was coated at 5 μg/mL with 50 μL/well onto a Maxisorp plate and incubated at 4 °C overnight. The following day, the plates were washed 6 times with PBS/T, before blocking with 150 μL/well Casein Blocker at RT for 1 h. After washing again six times in PBS/T, PfRH5 protein variants were loaded (50 μL in triplicate) on to the plate at 4 dilutions in Casein Blocker (800, 200, 50 and 12.5 ng/mL) and incubated at RT for 2 h. After a further wash, plates were incubated with polyclonal anti-PfRH5 rabbit serum^7^ diluted 1:1000 in Casein Blocker, using 50 μL/well at RT for 1 h. After a further wash, plates were incubated with goat anti-rabbit IgG alkaline phosphatase diluted 1:5000 in Casein Blocker, using 50 μL/well at RT for 1 hour. After a final six washes in PBS/T, followed by two washes in PBS, plates were developed as for the anti-PfRH5 quantification ELISA and optical density read at 405 nm.

### Surface plasmon resonance (SPR)

The production of recombinant basigin in Origami B (DE3) *E. coli* has been previously described^40^. A section of the basigin gene encoding immunoglobin domains 1 and 2 of the short isoform (αα 22–205) was cloned with an N-terminal His6 tag followed by a tobacco etch virus (TEV) protease cleavage site. TEV cleavage leaves an additional glycine at the N-terminus from the cleavage site. SPR experiments were carried out using a BIAcore T200 instrument (GE Healthcare). Experiments were performed at 20 °C in 10 mM HEPES (pH 7.4), 150 mM NaCl, 3 mM EDTA, 0.005% Tween-20, 2 mg/mL dextran, and 1 mg/mL salmon sperm DNA (Sigma). Basigin was immobilized on a CM5 chip (GE Healthcare) by amine coupling (GE Healthcare kit) to a total of 850 Response Units (RU). A concentration series of each PfRH5 variant protein (8, 4, 2, 1, 0.5, 0.25, 0.125 and 0.0625 μM) was injected over the basigin-coated chip for 120 s at 30 μL/min, followed by a 300 s dissociation time. The chip surface was then regenerated with 30 s of 2 M NaCl. Specific binding of the PfRH5 protein was obtained by subtracting the response from a blank surface from that of the basigin-coated surface. The kinetic sensorgrams were fitted to a global 1:1 interaction model, allowing determination of the dissociation constant, K_D_, using BIAevaluation software 1.0 (GE Healthcare).

### Free thiol assay

Free sulfhydryl groups were estimated using Ellman’s reagent^57^ and a DL-Dithiothreitol standard. PfRH5 samples were prepared in the presence of 6 M guanidine-HCl which denatures the protein but keeps disulfide bonds intact.

### Rabbit immunization

Rabbit work was approved by the University of Oxford Animal Welfare and Ethical Review Body, and performed in accordance with all applicable regulations. Rabbit protein immunizations were carried out by Biogenes (Germany). ZiKa rabbits (*n* = 4/group) were immunized intramuscularly (i.m.) with 20 μg protein on day 0 formulated in complete Freund’s adjuvant, followed by two booster immunizations i.m. on days 28 and 56 with the same dose of protein formulated in incomplete Freund’s adjuvant. Serum was collected two weeks after the final immunization on day 70 and shipped frozen. In a separate group, four rabbits were immunized with viral vectors encoding full-length PfRH5 (similar to version 4.0 sequence, Fig. S3B) and described previously^7^. Rabbits were primed i.m. with 2.5 × 10^8^ infectious units (ifu) human adenovirus serotype 5 (AdHu5) encoding the antigen, and boosted i.m. 8 weeks later on day 56 with 5 × 10^7^ pfu modified vaccinia virus Ankara (MVA) encoding the same antigen; serum was harvested on day 70. Sera were also used from a historical study, reported previously^16^. In this case, two New Zealand white rabbits were immunized at Agrobio (France) and three Zika rabbits were immunized at Biogenes (Germany) with exactly the same viral vectors, doses and regime and serum harvest between days 71–77.

### Anti-PfRH5 Serum IgG ELISA

To measure anti-PfRH5 serum IgG responses by ELISA, PfRH5 v2.0 protein was used that lacked the C-terminal His6 tag–this removed the possibility of detecting anti-His6 responses. This v2.0 protein was generated in S2 cells subsequent to the work described here and will be described elsewhere [Jin *et al*., in preparation]. Rabbit IgG ELISAs were carried out using a standardized ELISA according to previously described methodology^58^,^59^ and reagents^7^, and using a reference sample generated from high-titer sera from a viral vectored immunized rabbit^7^. A 1:3600 dilution of the reference sample gave an OD_405_ = 1.0, and thus this reference serum was taken to be 3600 arbitrary units (AU). Test samples were diluted appropriately so that their optical density 405 nm (OD_405_) could be read off the linear part of the reference curve.

In order to convert the responses in AU to μg/mL, total IgG was isolated from six pools of 5 mL serum (4 rabbits per pool and including a control pool of 4 naïve/pre-bleed sera) using protein G (Pierce Biotechnology, Rockford, IL, USA) packed into disposable 10 mL columns (Pierce) according to the manufacturer’s instructions. The eluted fractions were immediately neutralized with Tris buffer (pH 9.0), dialyzed against PBS, and concentrated with centrifugal filter devices to a concentration of 28–34 mg/mL. PfRH5-specific antibodies were isolated from total IgG using PfRH5 v2.0 protein lacking the His6 tag [Jin *et al*., in preparation] in PBS coupled with NHS-activated sepharose resin (GE Healthcare Life Sciences, Buckinghamshire, UK) packed into disposable 10 mL columns (Pierce) according to the manufacturer’s instructions. Each of the six pools was purified on a separate column, packed with 0.5 mg PfRH5 protein coupled to 0.5 mL sepharose resin (1 mL slurry). For each pool, 5 mg purified total IgG were loaded onto the column. The eluted fractions were immediately neutralized with Tris buffer (pH 9.0), dialyzed against PBS, and concentrated with 30 kDa-membrane Amicon Ultra-15 Centrifugal Filter Units (Merck-Millipore, Darmstadt, Germany) to approximately 0.3 mL. The concentration of PfRH5-specific rabbit IgG in eluted fractions was measured by absorbance at 280nm using NanoDrop (NanoDrop, Wilmington, DE, USA), and confirmed by ELISA (see below). For each eluted fraction, the anti-PfRH5 response in AU was also measured using the above protocol. The PfRH5-specific IgG concentration of each rabbit pool was subsequently plotted against its PfRH5 ELISA AU and linear regression was performed. The fit was satisfactory (R^2^ = 0.987) and the slope of the regression line was used to convert the ELISA AU of PfRH5 vaccinated rabbit sera into concentration of RH5-specific IgG. The conversion factor (slope) is 0.434 μg/mL PfRH5-specific rabbit IgG per ELISA AU.

### Rabbit IgG Concentration ELISA

Rabbit IgG concentration ELISAs were measured using a standardized ELISA according to previously described methodology^58^,^59^ and reagents^7^, with the following exceptions: IgG was captured by coating plates with 50 μL/well of 2 μg/mL unlabelled goat anti-rabbit IgG Fab (H+L) (Southern Biotech) and left covered overnight at 4 °C. A standard curve was generated by serially diluting a sample of rabbit total IgG of known concentration, as measured by NanoDrop. This sample was diluted to 2 μg/mL and subsequently diluted 2-fold serially to obtain a 10-point standard curve. Plates were blocked with 5% skimmed milk and the secondary detection antibody was goat anti-rabbit IgG (whole molecule)-alkaline phosphatase (Sigma).

### Assay of growth inhibition activity (GIA)

Total IgG was purified from rabbit sera using protein G columns (Pierce). The *P. falciparum* 3D7 clone was maintained in continuous culture using fresh O+ erythrocytes at 2% hematocrit and synchronized by two incubations in 5% sorbitol 6–8 h apart. Synchronized trophozoites were adjusted to 0.3% parasitaemia and then incubated for 42 h with the various IgG concentrations. Final parasitemia was determined by biochemical determination of parasite lactate dehydrogenase^44^. Percentage growth inhibition is expressed relative to wells containing IgG from control immunized rabbits^7^. The mean of the three replicate wells was taken to obtain the final data for each individual rabbit at each tested IgG concentration. Experiments were performed twice with very similar results.

### Statistical analysis

Data were analyzed using GraphPad Prism version 5.04 for Windows (GraphPad Software Inc., California, USA). For the non-linear least squares regression, the equation: Y = Bottom + (Top − Bottom)/(1 + 10 ^ ((LogEC_50_ − X) * HillSlope)) was used with four parameter curve and log_10_ transformed ELISA data, constrained at the top to <100% and at the bottom to >0% GIA.

## Additional Information

**How to cite this article**: Hjerrild, K. A. *et al*. Production of full-length soluble *Plasmodium falciparum* RH5 protein vaccine using a *Drosophila melanogaster* Schneider 2 stable cell line system. *Sci. Rep.* **6**, 30357; doi: 10.1038/srep30357 (2016).

## Supplementary Material

Supplementary Information

## Figures and Tables

**Table 1:** PfRH5 protein purification steps and process yield.

Purification step	Protein recovered (mg)	Step yield (%)	Process yield (%)
1.25 L PfRH5 v1.0 S2 supernatant	37.5	100	100
TFF, buffer exchange, and filtration	30	80	80
Ni-IMAC elution	3.6	12	10
Con A 4B flow through	3.0	83	8
SEC and concentration	1.6	53	4

Representative data for the amount of PfRH5 v1.0 protein recovered at each step of the purification process, along with the step yield and overall process yield. Protein recovery was monitored using an anti-PfRH5 quantification ELISA.
