# Supplementary material for: Production of full-length soluble Plasmodium falciparum RH5 protein vaccine using a Drosophila melanogaster Schneider 2 stable cell line system
Source: Sci Rep. 2016 Jul 26;6:30357. doi: 10.1038/srep30357 (PMC4960544; doi:10.1038/srep30357)
Supplement: Supplementary Information [file srep30357-s1.pdf]

Production of full-length soluble *Plasmodium falciparum* RH5 protein vaccine using a *Drosophila melanogaster* Schneider 2 stable cell line system

Kathryn A. Hjerrild[1], Jing Jin[1], Katherine E. Wright[2], Rebecca E. Brown[1], Jennifer M. Marshall[1], Geneviève M. Labbé[1], Sarah E. Silk[1], Catherine J. Cherry[1], Stine B. Clemmensen[3], Thomas Jørgensen[3], Joseph J. Illingworth[1], Daniel G. W. Alanine[1], Kathryn H. Milne[1], Rebecca Ashfield[1], Willem A. de Jongh[3], Alexander D. Douglas[1], Matthew K. Higgins[2*], and Simon J. Draper[1*].

[1] The Jenner Institute, University of Oxford, Old Road Campus Research Building, Oxford, OX3 7DQ, UK.

[2] Department of Biochemistry, University of Oxford, South Parks Road, Oxford, OX1 3QU, UK.

[3] ExpreS^{2}ion Biotechnologies, SCION-DTU Science Park, Agern Allé 1, Hørsholm DK-2970, Denmark.

* Corresponding authors.

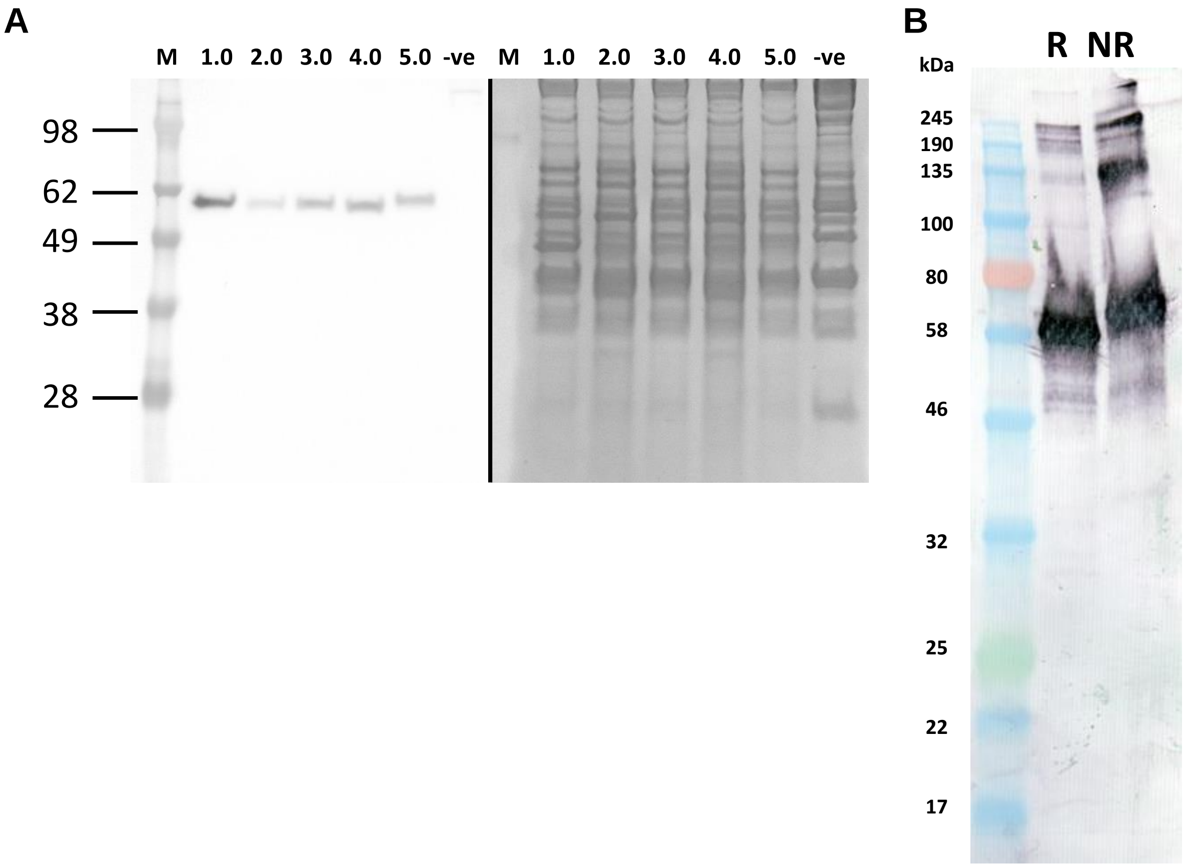

Figure S1. Assessment of *Drosophila* S2 cells expressing PfRH5 protein variants.

(A) Transient transfections, expressing PfRH5 protein variants versions 1.0 – 5.0, were performed. 30 µL samples of culture supernatants were run from batch cultures harvested on day 4. SDS-PAGE gels were run under reducing conditions and stained for total protein (right hand side) or Western blots performed with anti-Penta-His mAb (left hand side). Results are shown for each variant. M = molecular weight markers. –ve = S2 cell supernatant control. **(B)** Samples of supernatant from the v1.0 PfRH5 stable cell line were run under reducing (R) and non-reducing (NR) conditions. The Western blot was stained with anti-PfRH5 polyclonal rabbit sera.

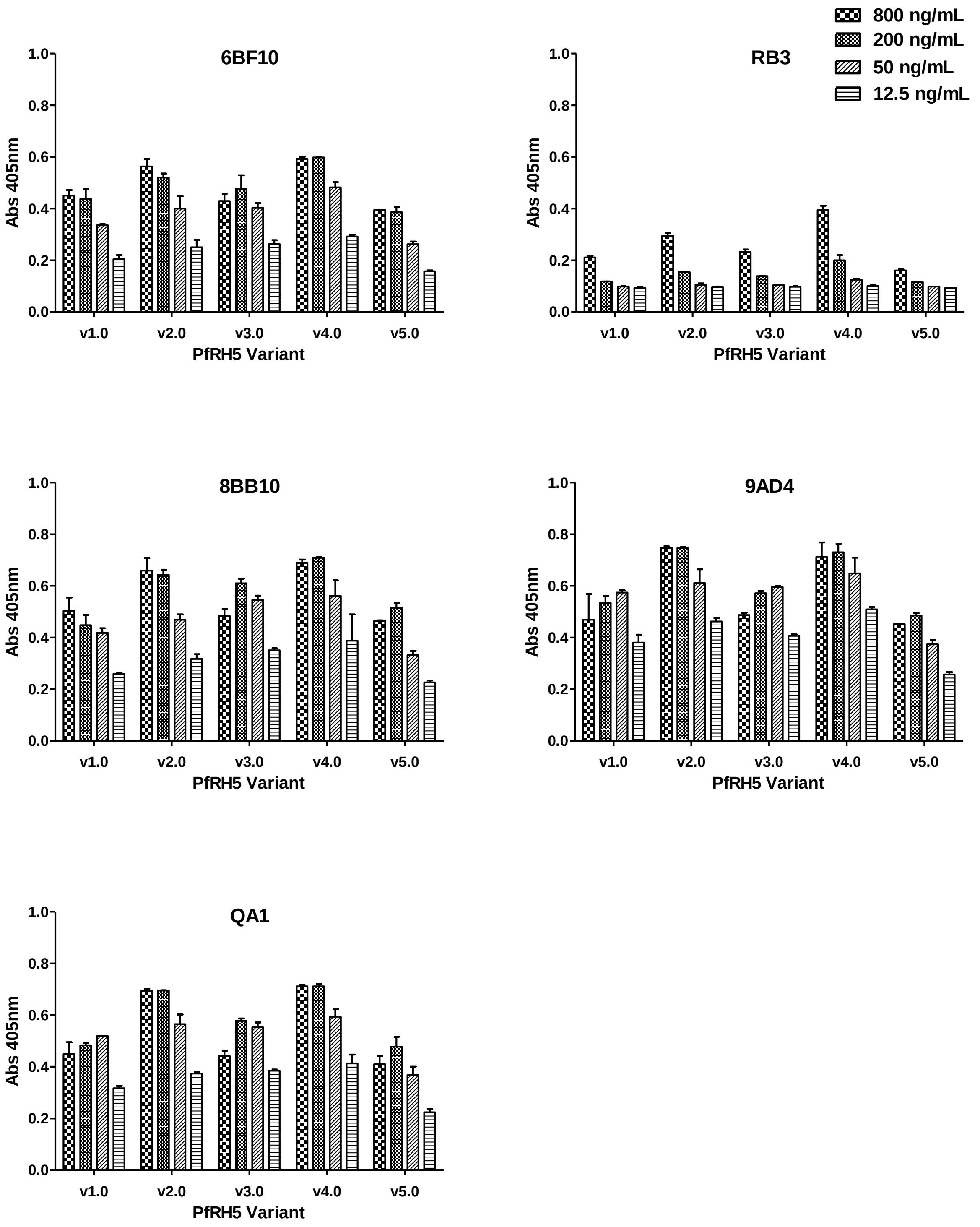

Figure S2. Capture ELISAs using PfRH5-specific mAbs.

Capture ELISA using a panel of PfRH5-specific mAbs. Each mAb (6BF10, RB3, 8BB10, 9AD4 and QA1) was coated to the plate at a concentration of 5 μg/mL, and each PfRH5 protein variant was tested for binding using a dilution series of protein ranging from 800 ng/mL to 12.5 ng/mL. Each sample was tested in triplicate for each concentration. Bars show the median plus range.

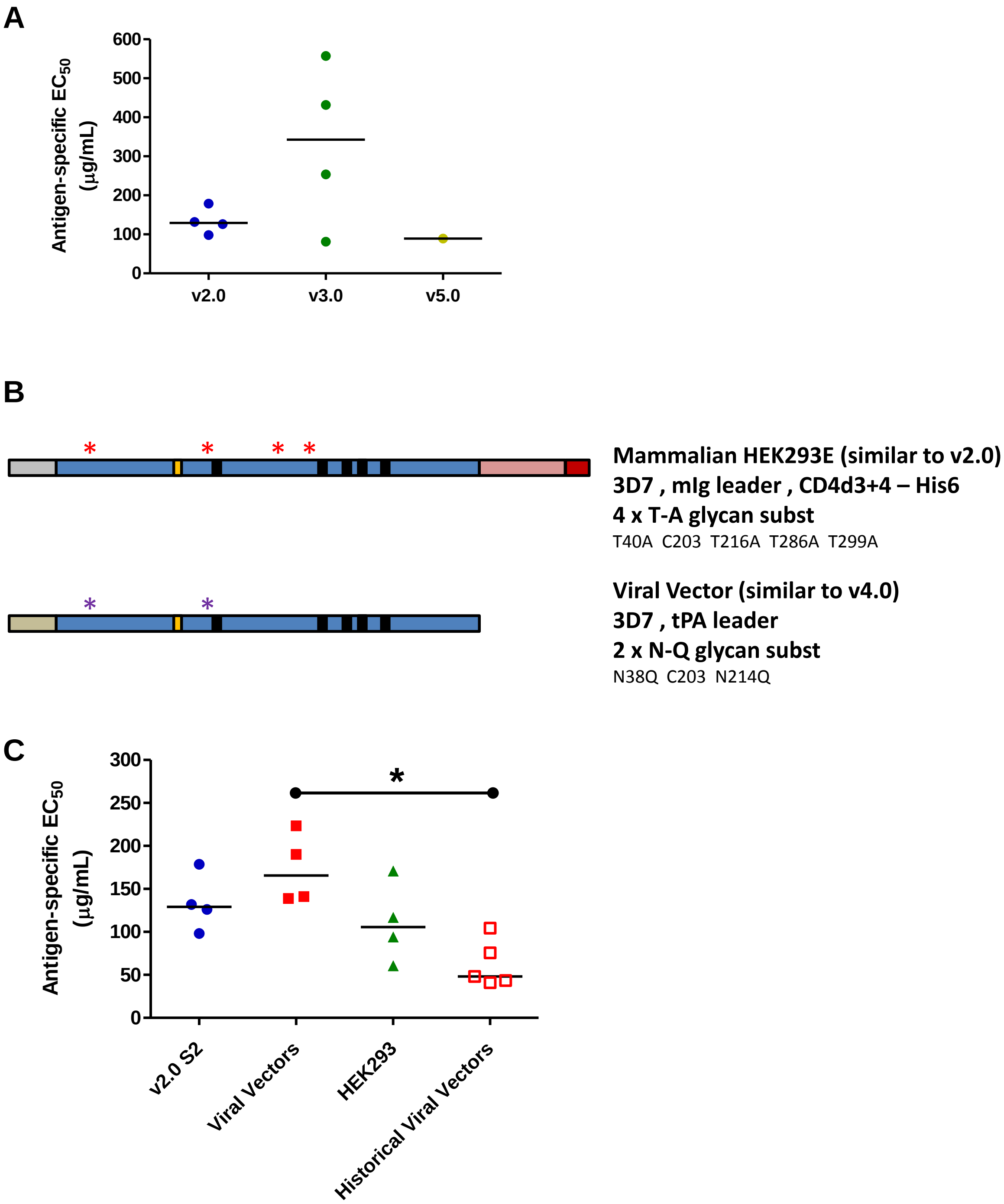

Figure S3. Qualitative assessment of PfRH5 vaccines.

(A) The antigen-specific EC_{50} was interpolated from the GIA data for each individual rabbit immunized with PfRH5 protein variants produced in S2 cells. This was calculated for nine of the rabbits that reproducibly achieved >40% GIA in the original assay (Fig. 5A). Individual and median results are plotted. **(B)** Schematic of PfRH5 protein variants expressed from the viral vectored

vaccines and a PfRH5 protein variant produced in HEK293E cells. The viral vectored construct includes a human tissue plasminogen activator (tPA) leader sequence followed by the equivalent of the PfRH5 v4.0 sequence (E26-Q526) codon optimized for mammalian expression, with no C-terminal tags. The mammalian cell produced PfRH5-Cd4-His6 protein includes a murine Igκ leader sequence (mIg) followed by the equivalent of the PfRH5 v2.0 sequence (F25-Q526) codon optimized for mammalian expression, with C-terminal tags – rat CD4 domains 3 and 4 (CD4d3+4) followed by His6 [1]. **(C)** The antigen-specific EC_{50} was interpolated from GIA data for each individual rabbit as in panel (A). Data show the results for PfRH5 protein v2.0 produced in S2 cells, as well as the viral vector and HEK293 cell-produced protein outlined in panel (B). Sera were also re-analyzed from a historical study [2] where five rabbits were immunized in exactly the same manner with the viral vectors. Individual and median results are plotted.

Supplementary References

1 Douglas, A. D. *et al.* A PfRH5-Based Vaccine Is Efficacious against Heterologous Strain Blood-Stage Plasmodium falciparum Infection in Aotus Monkeys. *Cell Host Microbe* **17**, 130-139 (2015).

2 Williams, A. R. *et al.* Enhancing Blockade of Plasmodium falciparum Erythrocyte Invasion: Assessing Combinations of Antibodies against PfRH5 and Other Merozoite Antigens. *PLoS Pathog* **8**, e1002991 (2012).
